# Preoxidation-assisted nitrogen enrichment strategy to decorate porous carbon spheres for catalytic adsorption/oxidation of methyl mercaptan†

**DOI:** 10.1039/d0ra07375j

**Published:** 2020-10-12

**Authors:** Changming Zhang, Yaqi Wang, Xiaochao Zhang, Rongxian Wang, Lifang Kou, Rui Li, Caimei Fan

**Affiliations:** College of Mining Engineering, Taiyuan University of Technology Taiyuan 030024 PR China zhangchangming@tyut.edu.cn +86 351 6111190 +86 351 6010551; College of Chemistry and Chemical Engineering, Taiyuan University of Technology Taiyuan 030024 PR China zhangxiaochao@tyut.edu.cn; Key Laboratory of Coal Science and Technology, Ministry of Education and Shanxi Province Taiyuan 030008 PR China

## Abstract

Porous carbon spheres with high surface area and microporous structure were synthesized from alkyl phenols and formaldehyde *via* suspension polymerization and steam activation. The effects of air oxidation and ammonia solution heat treatment on the pore structure and surface chemistry of the carbon spheres were studied for catalytic oxidation of CH_3_SH. The structure property and surface chemistry of the obtained carbon spheres were characterized by N_2_ adsorption–desorption, FTIR, scanning electron microscopy, XRD, elemental analysis, X-ray photoelectron spectroscopy and Boehm titration, and then thermal analysis and gas chromatography-mass spectrometry were applied to investigate the catalytic oxidation product. Results show that the as-prepared microporous carbon spheres through direct ammonia treatment have a high surface area value of 1710 m^2^ g^−1^ and a total pore volume of 0.83 cm^3^ g^−1^. Moreover, the preoxidation-assisted nitrogen enrichment strategy not only increases the surface area and total pore volume of the carbon spheres, but also introduces more active nitrogen species such as pyridinic nitrogen and quaternary nitrogen, leading to the highest nitrogen content of 7.13 wt% and the highest CH_3_SH capacity of 622.8 mg g^−1^ due to the pyridinic nitrogen and quaternary nitrogen as function of catalysts. In addition, water and oxygen have a beneficial effect on CH_3_SH oxidation over the nitrogen modified carbon spheres, and the basic oxidation product is CH_3_SSCH_3_ that can be further oxidized into CH_3_SO_2_SCH_3_ according to DTG and GC/MS analysis. The great recycling stability after ten cycles with a reserved CH_3_SH capacity of 97% demonstrates that the porous carbon spheres obtained by preoxidation-assisted enriched nitrogen strategy are promising for catalytic oxidation of CH_3_SH.

## Abstract 2

Porous carbon spheres with high surface area and microporous structure were synthesized from alkyl phenols and formaldehyde *via* suspension polymerization and steam activation.

## Graphical abstract

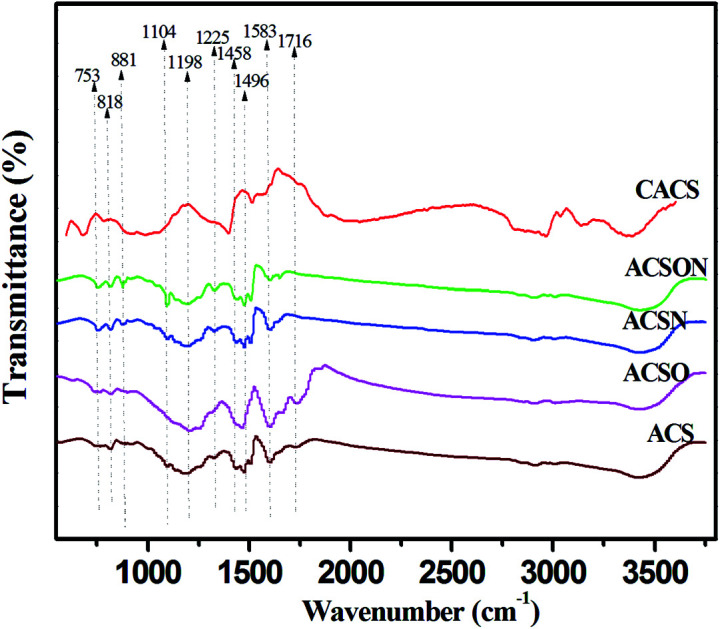

## 1. Introduction

It is well known that methyl mercaptan (CH_3_SH) widely results from many chemical and agricultural processes related to natural gas, petroleum gas, coal gas and so on.^1–4^ Once CH_3_SH is released into the atmosphere, it will give rise to the formation of sulfate particles, further resulting in environment problems such as acid rain.^5–7^ As a result, it is considerably important to carry out desulfurization of liquids and gases. More research activities have been developed to remove CH_3_SH efficiently. For instance, the mature commercial technology is hydrodesulfurization that has been widely used in the traditional refineries with good removal results, but it has serious drawbacks such as high hydrogen consumption and energy requirements.^8,9^ Therefore, extensive research has been carried out to propose alternative technologies focused on convenient conditions without hydrogen.

These alternative technologies are related to adsorption, decomposition, catalytic adsorption/oxidation, and photocatalytic oxidation.^10–14^ Catalytic adsorption/oxidation has been proposed as a promising method for CH_3_SH removal by using solids adsorbents.^15–17^ Among them, nitrogen-doped carbon materials are thus among the most promising candidates for enhancing adsorption applications.^18–20^ For example, Bagreev^21^*et al.* impregnated different activated carbons with urea followed by thermal treatment at 450 and 950 °C, and the CH_3_SH adsorption capacity increased around 10 times after introduction of nitrogen species that can transport electron from sulfur to oxygen as a function of catalysts. Shi^22^*et al.* proposed that doping N into the carbon nanotube (CNT) is beneficial for the adsorption of S compounds through either polar or acid–base interactions, and pyridinic N and pyrrolic N improve the effects of S interaction with CNT and the capacitance, respectively. Liu^23^*et al.* prepared nitrogen-doped coconut shell activated carbon catalysts for the removal of CH_3_SH by impregnating urea or melamine with subsequent heat treatment. The results showed that the CH_3_SH removal capacity increased with the nitrogen content, especially pyridinic nitrogen and quaternary nitrogen. Moreover, the exhausted nitrogen-rich activated carbon can be easily regenerated with a reserved CH_3_SH capacity of 88.33% after four cycles.

Porous carbon spheres as one kind of the carbon-based materials show many charming advantages over powdered and granular activated carbon, such as suitable ball size, smoother surface, better fluidity, high mechanical strength, and high adsorption capacity,^24,25^ leading to the widespread technological applications in chemical protective clothing, blood purification, catalyst support and adsorption processes.^26–28^ Various polymers such as petroleum pitch, sulfonated poly(styrene-divinylbenzene) and phenolic resins have been mainly adopted to prepare porous carbon spheres by carbonization and activation. Romero-Anaya^29^*et al.* obtained the carbon spheres from petroleum pitch by CO_2_ or steam activation with high microporosity and low surface oxygen groups content that show a positive influence on the adsorption application of non-polar volatile organic compounds (VOCs) at low concentrations, and thus leading to toluene adsorption capacities as high as 46 g toluene/100 g carbon spheres. Wickramaratne^30^*et al.* synthesised phenolic resin spheres with diameters from 200 to 420 nm by the one-pot modified Stöber method, and the high CO_2_ adsorption capacities reached 4.55 and 8.05 mmol g^−1^ on these AC spheres at 1 bar and two temperatures, 25 and 0 °C, respectively. Sun^31^*et al.* prepared activated carbon beads with desirable spherical forms from phenolic resins using a novel hydrothermal process coupled with a range of post-preparation treatments, and found that the well-developed porous system is important to obtain a higher CO_2_ capture capacity at both ambient and elevated pressures. Sulfur-doped microporous carbon spheres were synthesized from poly(styrene-divinylbenzene) through the sulfonation, carbonization, and KOH activation for CO_2_ adsorption, and the CO_2_ uptake presents 4.21 mmol g^−1^ and 2.54 mmol g^−1^ at both 25 °C and 50 °C under ambient pressure, respectively. The high capacity has been attributed to abundant ultramicropores and high contents of oxidized sulfur functional groups.^32^

What's more, phenolic resins are considered as promising polymeric precursors to prepare high surface area and desired pore structure of carbon spheres for their low level of inorganic impurities and negligible ash content.^33,34^ Resin based carbon spheres with high surface area and different porous structure were prepared by adjusting two organic additives such as ethylene glycol and polyethylene glycol for dibenzothiophene adsorption in our previous works,^24^ and the carbon spheres were proposed as promising adsorbents for deep desulfurization of liquid hydrocarbons. In the present work, we firstly prepared resin spheres from mixture of isopropylphenol and formaldehyde by suspension polymerization, and then obtained carbon spheres with high surface area and microporous structure from these resin spheres *via* steam activation. Furthermore, the effects of air oxidation and ammonia solution heat treatment on the pore structure and surface chemistry of carbon spheres were investigated for efficient catalytic oxidation of CH_3_SH. Moreover, N_2_ adsorption–desorption, Fourier transform infrared spectroscopy (FT-IR), X-ray diffractometer (XRD), scanning electron microscope (SEM), elemental analysis, X-ray photoelectron spectroscopy (XPS) and Boehm titration were applied to characterize samples, and then thermal analysis (DTG) and gas chromatography-mass spectrometry (GC/MS) were carried out to investigate the created oxidation product in the process of catalytic oxidation. In addition, the stability and regeneration activity were evaluated by cycling tests. Finally, the possible mechanism of adsorption/oxidation of CH_3_SH over modified carbon spheres was discussed according to the experiment and characterization results.

## 2. Experimental

### 2.1 Materials

Isopropylphenol (99%), formaldehyde (37 wt% in water), triethanolamine, poly(vinyl alcohol), hexamethylene tetraamine and ammonia absolute (25 wt% in water) were purchased from Tianjin Tianli Chemical Corp. All solvents and other chemicals were AR grade and utilized without further purification.

### 2.2 Preparation of porous carbon spheres

Series of millimeter porous carbon spheres were prepared from alkyl phenol and formaldehyde according to the recipe reported previously.^24,25^ Firstly, resin spheres were prepared from isopropylphenol and formaldehyde by suspension polymerization. In a typical synthesis, the polymerization reaction was carried out in a 1000 mL round-bottomed four-neck reaction vessel with a Teflon stirrer, a reflux condenser, and a thermocouple. The isopropylphenol was polymerized with an aqueous solution (37–41%, w/v) of formaldehyde in a molar ratio of 1 : 5 in the presence of triethylamine (TEA, 1.6 wt%), followed by dispersing the resulting mixture into 300 mL of deionized water *via* stirring at 600 rpm. Then, poly(vinyl alcohol) (PVA, 7.5 wt%) was added to the above mixture at 96 °C by stirring at 600 rpm for 30 min. Afterwards, hexamethylene tetraamine (HMTA, 6.0 wt%) was added to reaction vessel and polymerization reaction was carried out at the same temperature and fixed agitation rate (600 rpm) for 4 h. After cooling to room temperature, resin spheres were obtained *via* solid–liquid separation. And then they were further carbonized at 850 °C for 45 min under nitrogen atmosphere and activated at the same temperature with steam for 1.5 h. The obtained carbon spheres will be denoted as ACS. Furthermore, the prepared ACS was oxidized by dry air with a flow rate of 60 mL min^−1^ for 2 h at 350 °C, and denoted as ACSO.

The ACS and ACSO were separately further functionalized by heat treatment with ammonia solution. In a typical run, the sample was placed in a vertical cylindrical furnace under nitrogen flow rate of 60 mL min^−1^, the temperature was increased with a ramp rate of 3 °C min^−1^. When the temperature attained 800 °C, the ammonia solution was introduced at a flow rate of 10 mL min^−1^ for 1 h, and then cooled down to room temperature. The obtained samples were named as ACSN and ACSON, respectively. In addition, the commercialized activated carbon spheres (CACS) was purchased from Taiyuan New Process Technology Corp (Shanxi, China) for comparison.

### 2.3 Characterization

Materials characterizations were carried out reference to our previous publications.^26,27^ The textural parameters of all the studied ACSs were carried out by N_2_ adsorption/desorption at −196 °C using a ASAP 2020 instrument. The samples were separately degassed at 250 °C in a vacuum environment for a period of at least 4 h prior to measurements. Experimental adsorption data at the relative pressure (*P*/*P*_0_) less than 0.3 was used to calculate surface area values using the standard Brunauer, Emmett, and Teller (BET) equation. The pore size distribution (PSD) was determined by applying density functional theory (DFT) method based on nitrogen adsorption data. FT-IR spectra of activated carbon samples were obtained utilizing a PerkinElmer Spectrum 100 FT-IR spectrometer. A disk made of pure KBr was used as a reference sample for background measurements. The carbon spheres–KBr mixtures at a ratio of 1 : 300 were ground in an agate mortar and then pressed under vacuum conditions in a hydraulic press. Before the spectrum of a sample was recorded, the background line obtained was arbitrarily and automatically subtracted. The spectra were recorded from 4000 to 400 cm^−1^ at a scan rate of 0.2 cm s^−1^, and the number of interferograms with a nominal resolution of 4 cm^−1^ was fixed at 100. The spectra were recorded in the range of 400–4000 cm^−1^ at a resolution of 4 cm^−1^. The surface morphology of the samples was observed on a Hitachi S-4800 field emission scanning electron microscope (SEM) operating at 3 kV. The crystallinity information on the surface of samples was determined by a X-ray diffraction-meter (APLX-DUO, BRUKER, Germany, XRD) using a Cu Kα radiation source (*λ* = 1.5406 Å, 35 kV voltage, 30 mA electric current and 2*θ* range from 10° to 90° at 5° min^−1^ scanning speed). Surface chemistry was evaluated using the Boehm titration method. For the purpose of this research, 1 g of carbon sample was placed in 50 mL of 0.05 N sodium hydroxide or hydrochloric acid. The vials are sealed and shaken for 24 h and then 10 mL of each filtrate was pipetted and the excess of base or acid was titrated with HCl or NaOH. The numbers of acidic sites were calculated under the assumption that NaOH neutralizes acidic groups and HCl neutralizes basic groups. The chemical composition of the activated carbon samples was measured by an Elementar Vario Macro EL Cube microanalyzer. XPS was measured on a PHI5300 X-ray photoelectron spectrometer. Monochromatic Al Kα source (1486.6 eV) was used at a power of 210 W. The resolution of the instrument is 0.55 and 0.70 eV for Ag 3d and Au 4f peaks, respectively. Survey scans were collected for binding energy ranging from 1100 eV to 0 with an analyzer pass energy of 160 eV with a step of 0.6 eV for a dwell time of 150 ms. For the high-resolution spectra, the pass-energy was 20 eV with a step of 0.1 eV and dwell time of 200 ms. 0.4 g carbon powder was placed in 20 mL of water and equilibrated during the night. Then the pH of the suspension was measured by a pH meter (OAKLON, PC700) for comparison. For exhausted samples, the pH is denoted as pHE. 1 mL methanol and 0.5 mL carbon powder were mixed in a flask and warmed at 60 °C for 1 h. Then, the suspension liquid was analyzed by gas chromatography-mass spectrometry (GC/MS) experiments using a PerkinElmer gas chromatograph/mass spectrometer.

### 2.4 Adsorption test and catalytic test

**Scheme 1:**
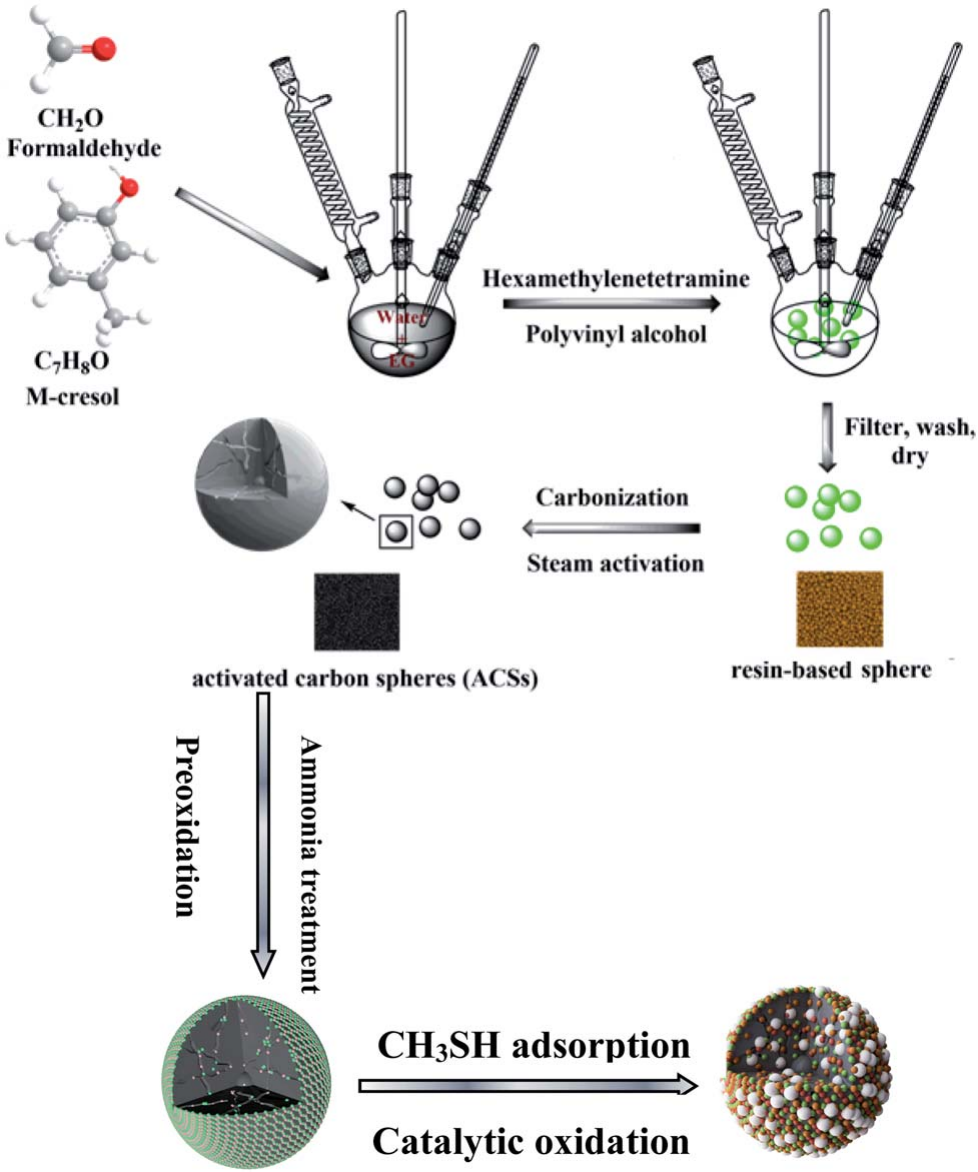
Schematic route for catalytic adsorption/oxidation of CH_3_SH over the nitrogen enriched porous carbon spheres.

Dynamic tests were carried out at 25 °C to evaluate the capacity of the sorbents for CH_3_SH removal under wet conditions.^18^ Adsorbent samples were ground and packed into a glass column, and prehumidified with moist air (relative humidity 80% at 25 °C) for 1 h. The amount of water adsorbed was estimated from the increase in the sample weight. Moist air (relative humidity 80% at 25 °C) containing 0.3% (3000 ppm) CH_3_SH was then passed through the column of adsorbent at 0.5 L min^−1^. The breakthrough of CH_3_SH was monitored using a Micromax monitoring system with an electrochemical sensor calibrated with CH_3_SH. The test was stopped at the breakthrough concentration of 20 ppm. After the adsorption test, the exhausted samples were designated with the letter E. To study the roles of water and oxygen in the removal of CH_3_SH, contrast experiments were also tested using dry air and dry nitrogen as carrier gases for ACS and ACSON, and denoted as ACS-A, ACS-N, ACSON-A, and ACSON-N, where A and N represent dry air and dry nitrogen, respectively. The adsorption capacities of each sorbent in terms of mg of CH_3_SH per gram of carbon were calculated by integration of the area above the breakthrough curves, and from the CH_3_SH concentration in the inlet gas, flow rate, breakthrough time, and mass of sorbent. In addition, the process of regeneration of exhausted samples was carried out as follows, 6 mL exhausted sample was mixed with 300 mL of ethanol for 5 h at 50 °C and then filtered. Ten cycles were performed, and then the carbon sample was heated at 500 °C for 1 h under a nitrogen atmosphere. Finally denoted as ACSON-10 (Scheme 1).

## 3. Results and discussion

### 3.1 N_2_ adsorption–desorption analysis

The N_2_ adsorption–desorption isotherms were measured at −196 °C to investigate the porous structure property of the untreated carbon spheres and modified samples, and the result curves are shown in Fig. S1.† It can be seen from the curves that the isotherms of all the samples present similar features, and can be related to the type I according to the Brunauer–Deming–Deming–Teller (BDDT) classification, which show an abrupt knee at low relative pressure and tend to balance after *P*/*P*_0_ > 0.4 without obvious hysteresis loops in Fig. S1(a),† indicating the main micropore structure of the carbon spheres.^25,26^ Furthermore, all of the carbon spheres show pore size distribution around 4 nm in Fig. S1(b),† and have a concentrated pore size distribution in the range of 0.5–1 nm, which is the characteristic of micropore carbon materials. The characteristic is well accordant with the N_2_ adsorption/desorption isotherms.

It is well known that the porous structure parameters such as BET surface area, total pore volume and micropore volume are favourable implements to study the pore characteristics, and the corresponding results are listed in Table 1. Compared to the virgin and commercial carbon spheres, it can be found that ammonia solution thermal treatment remarkably improves the porous structure of the virgin and oxidized carbon spheres, and leads to the increase in BET surface area, total pore and micropore volumes of ACSN and ACSON. The obvious changes of these parameters can be primarily attributed to the surface functional groups decomposition of pore channels under high temperature (800 °C) by creating. It is reported that the ammonia solution can decompose to generate the free radicals such as atomic hydrogen, NH_2_ and NH under high temperature (800 °C).^35,36^ Moreover, partial gasification reaction between the carbon spheres and the generated free radicals from the above thermal decomposition also makes a contribution to the porous development. Therefore, the highest surface area, total pore volume and micropore volume can attain 1710 m^2^ g^−1^, 0.83 cm^3^ g^−1^ and 0.74 cm^3^ g^−1^ for the direct ammonia treated sample ACSN, respectively. It can also been observed that the ammonia thermal treatment shows an evident contribution to the increase of micropore volume compared to the virgin carbon spheres, which can be ascribed to the above partial gasification reaction between the carbon spheres and the generated free radicals that exerts an active influence on the major microporosity domain by producing new micropore sites.^37,38^

**Table 1:** Porous structure parameters of the samples^a^

Sample	*S*_BET_ (m^2^ g^−1^)	*V*_total_ (cm^3^ g^−1^)	*V*_micro_ (cm^3^ g^−1^)	*V*_micro<1nm_ (cm^3^ g^−1^)	*V*_micro_/*V*_total_ (%)	*D*_p_ (nm)
ACS	1636	0.77	0.68	0.31	88.31	1.88
ACSO	1397	0.64	0.46	0.28	71.87	1.83
ACSN	1710	0.83	0.74	0.47	89.16	1.94
ACSON	1592	0.71	0.61	0.43	85.92	1.78
CACS	1447	0.67	0.45	0.22	67.16	1.85
ACSON-10	1523	0.69	0.56	0.39	81.15	1.81

a *V*_micro<1nm_ – volume of specific-micropores (pore diameter < 1 nm). *D*_p_ – the average pore size, determined by the equation of 4*V*_total_/*S*_BET_.

It can be seen that the BET surface are, total pore volume and micropore volume decrease in some degree after thermal air oxidization compared to the virgin carbon spheres. The results can be probably attributed to the partial collapse of some pore walls or the recession of some pore channels by the reaction related to O_2_, and eventually some micropores or/and mesopores became expanded or collapsed in different extent, resulting in the decrease of micropore volume.^39,40^ However, when the preoxidized carbon spheres are subjected to the ammonia thermal treatment under 800 °C, the oxygen surface groups firstly give rise to decomposition that creates the vacant sites in the pores, which provide more opportunities to increase reaction probability with created free radicals under the simultaneous ammonia treatment at 800 °C. It has been also reported that gasification with ammonia has a profitable to recover its porosity blocked by preoxidization, and increase the porous structure parameters over that of the oxidized sample.^41,42^ Moreover, the carbon spheres (ACSON) obtained by the preoxidation-assisted ammonia thermal treatment show bigger BET surface, total pore volume and microporous volume than those of the direct oxidized carbon spheres.

It can be observed from the data in Table 1 that the pore structure values of oxidized carbon spheres slightly decreased compared to the virgin carbon spheres and commercial carbon spheres. For instance, the BET surface area, total pore volume and microporous volume show 1397 m^2^ g^−1^, 0.64 cm^3^ g^−1^ and 0.46 cm^3^ g^−1^ after the oxidation, respectively. On the contrary, the BET surface area, total pore volume and microporous volume considerably become bigger after subsequent ammonia thermal treatment than those of the oxidized carbon spheres, and present 1592 m^2^ g^−1^, 0.71 cm^3^ g^−1^ and 0.61 cm^3^ g^−1^, respectively. Moreover, the carbon spheres have a higher micropore ratio (≥70%) except for CACS. In addition, the average pore size of all carbon spheres is less than 2 nm, which is in accordance with the characteristic of N_2_ adsorption/desorption. Therefore, the performance of these carbon spheres in catalytic oxidation of CH_3_SH may be primarily ascribed to the different surface chemical properties.

### 3.2 FTIR analysis

**Fig. 1:**
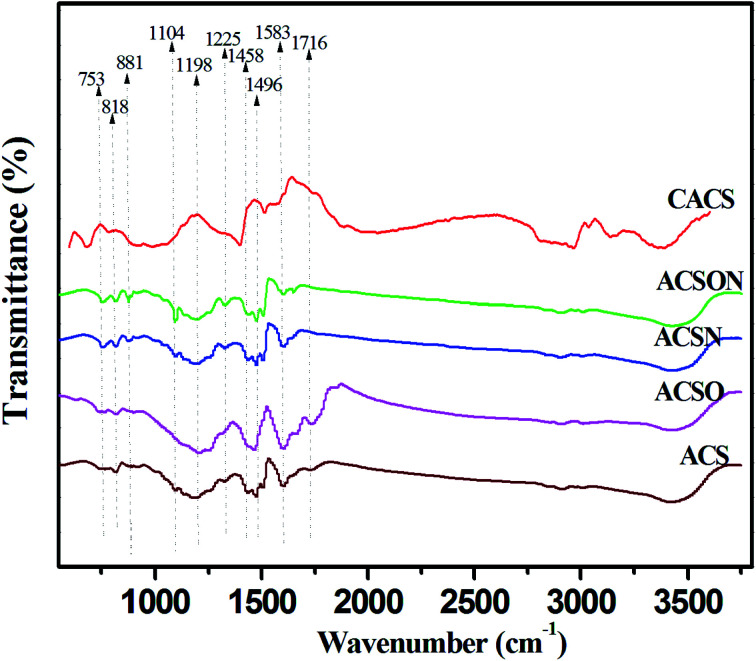
FTIR spectra of the samples.

The carbon spheres were subjected to the FTIR measurements in order to investigate the nature of the functional groups, and the FTIR analysis results of virgin carbon spheres and modified samples are shown in Fig. 1. It can be seen that several peaks located at the 3441, 2850, 1716, 1583, 1496, 1198 and 818 cm^−1^ appeared in the FTIR spectrum of virgin carbon spheres, which were related to O–H stretching vibration, –CH_2_– stretching vibration, C=O groups, C=C groups, aromatic ring, C–O stretching vibrations and C–H groups, respectively.^37,43^ It can be found that a sharp peak is located at 1716 cm^−1^ for oxidized carbon spheres ACSO compared to the ACS and other samples, which can be attributed to the carboxylic structure induced by thermal air oxidization.^25^ These oxygen surface groups can have a contribution to introduce the nitrogen containing functional groups into the carbon spheres during the subsequent ammonia treatment.

It can be observed that some changes have been created after ammonia thermal treatment in the spectra of ACSN and ACSON. Compared to the ACS and CACS, the new peaks can be seen at 1458, 1225 and 1104 cm^−1^, which can be corresponding to C=N stretching vibrations, C–N stretching vibrations and N–H stretching vibrations for all ammonia modified carbon spheres, respectively.^44,45^ Furthermore, another two obvious peaks at 881 and 753 cm^−1^ related to amine or amides functionalities can be found only in the in the spectra of ACSN and ACSON, which can be due to the formation of hydrogen bonds between the free radicals (such as NH_2_ and NH) and surface groups.^46,47^ These bands indicate that nitrogen containing functional groups can be effectively incorporated on the surface of carbon spheres by facile ammonia thermal treatment. It is noticeable that intensities of these bands related to the nitrogen containing functional groups are increased by the preoxidization-assisted ammonia thermal treatment compared to the direct ammonia modification. For instance, oxygen chemisorption, carboxylic species and cyclic anhydrides generated from preoxidization appear on the surface of carbon spheres, when the oxidized carbon spheres are subjected to the ammonia thermal treatment, the generated free radicals such as NH_2_ and NH can easily react with these oxygen functional groups to form amine or amide structures by dehydration reaction in the process of ammonia treatment, making a great contribution to the increase of bands intensity corresponding to these nitrogen containing functional groups.^37^ In addition, it can be seen that the peak related to carboxylic structure at 1716 cm^−1^ considerably diminished after ammonia thermal treatment, which can be due to the reaction that the generated free radicals give rise to attack to the surface oxides and active sites on the surface of carbon spheres to form nitrogen containing functional groups. And or decomposition of oxygen containing surface groups during higher temperature.^35^ Therefore, the band intensities related to nitrogen containing species exhibited an evident increase for oxidized carbon spheres following ammonia treatment compared to the direct ammonia treated carbon spheres, indicating that preoxidation-assisted ammonia treatment under high temperature significantly incorporates nitrogen containing species onto the surface of carbon spheres, which will be favorable for enhancement of CH_3_SH adsorption.

### 3.3 Surface morphology analysis

**Fig. 2:**
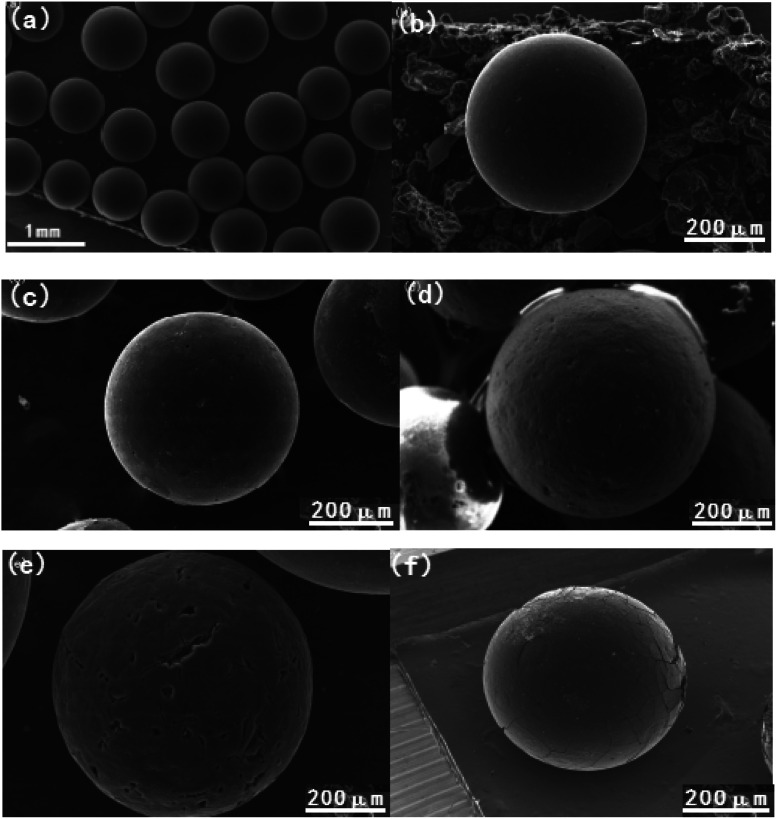
SEM images of (a and b) ACS, (c) ACSO, (d) ACSN, (e) ACSON and (f) CACS.

The surface morphology of the obtained activated carbon spheres were measured through SEM, and the SEM images are shown in Fig. 2. It can be seen that there are obvious differences in the surface morphology of all samples. For instance, the virgin carbon spheres ACS prepared from phenolic resin precursor by suspension polymerization and steam activation show millimeter scale sphere size, and present good sphericity and smooth surface as shown in Fig. 2(a) and (b), which can be ascribe to the favorable precursors of resin spheres and reasonable thermal condition of steam activation. The spherical precursors can provide uniform sphericity, while the steam activation has a profitable contribution to the development of pore structure. And then the surface of carbon spheres became a little rough with some small pores after air oxidation in Fig. 2(c), but the sphericity is also stable. These results can be due to the thermal air oxidation, in which the etching action between the oxygen molecules and the carbon surface or pore channel, leading to the CO_2_ departure, the pores expansion or collapse, and the formation of new oxygen containing groups.^25,40^ These consequences are consistent with the FTIR analysis and N_2_ adsorption. Furthermore, it can be observed that the surface of carbon spheres obtained by direct ammonia thermal treatment shows much more rough with stable sphericity than that of oxidized carbon spheres in Fig. 2(d). It is well known that NH_3_ molecules are smaller and easier to diffuse into the pore channels, and the created free radicals (NH_2_, NH) gradually reacted with the carbon surface or pore walls under high temperature, resulting in the surface etching and the expandation of pore structure or formation of new microporosity.^37^ As a result, the higher BET surface area and pore volume were shown for ACSN, and also generated obvious nitrogen containing groups in the FTIR spectrum. It is noticeable that the exterior surface of ACSON obtained by preoxidation-assistant ammonia thermal treatment shows considerable roughness and much big pores in Fig. 2(e), although it maintains good sphericity. The phenomenon can be attributed to the synergistic effect of thermal air oxidation and ammonia etching action,^37,39^ in which the surface oxygen functional groups to create vacant sites through thermal decomposition, and free radicals (NH_2_, NH and H) react with these vacant sites or surface oxygen functional groups to develop pore structure, leading to the increase of BET surface area and pore volume and simultaneously the introduction of nitrogen containing functional groups in the ACSON. On the contrary, CACS from commercial activated carbon is very different, the exterior surface shows some big cracks with roughness in Fig. 2(f), and the strength become a little poor.

### 3.4 XRD analysis

**Fig. 3:**
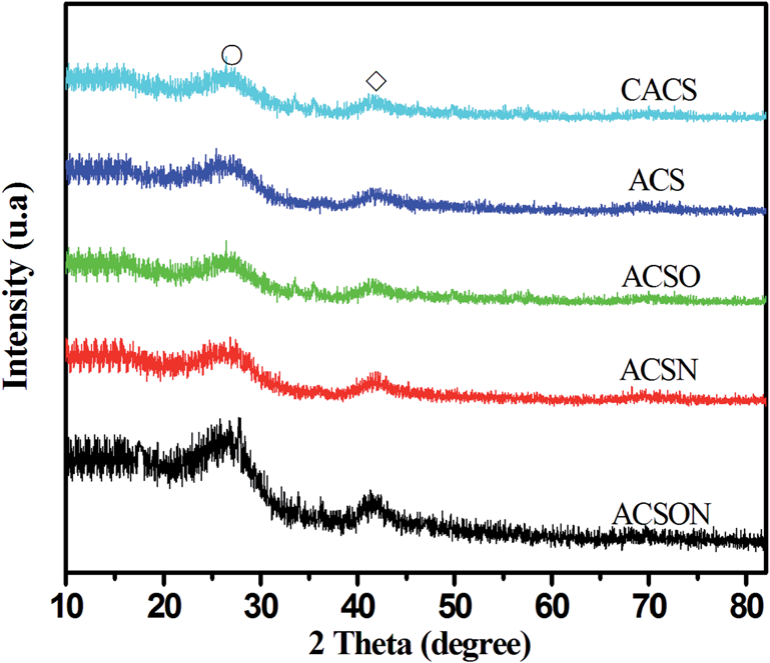
XRD spectra of the samples.

The as-obtained porous carbon spheres were subjected to the XRD measurement to investigate the crystal structure information, and the corresponding XRD analysis spectra were carefully recorded and shown in Fig. 3. It can be observed that two sharp diffraction peaks show at around 2*θ* = 26.58 °and 44.21° for all the prepared carbon spheres from the above XRD patterns, and no other obvious peaks appear in the spectra, which can be ascribe to the characteristics of porous carbon materials.^48,49^ In our work, the suspension polymerization is used to prepare the resin spheres precursor, afterwards, the resin spheres were carbonized and activated under high temperature with steam as a function of active agent that can react with the carbon. The primary reaction equations in the activation process are as follows to create the main carbon containing product, leading to the formation of the carbon network structure and the development of pore channel.

1 C + H_2_O → H_2_ + CO

2 CO + H_2_O → H_2_ + CO_2_

3 CO + 3H_2_ → CH_4_ + H_2_O

Furthermore, it has been proposed that the etching action of steam is generally completed accompanied by the decomposition of volatile components and the formation of new pores in the process of continuous C–H_2_O reaction.^50^ In our study, the primary component is carbon with small other composition, although the virgin carbon spheres were modified by different procedures. Therefore, the characteristics of XRD analysis spectra is highly consistent with reported porous carbon materials.

### 3.5 Boehm analysis

It has been proposed that the surface chemistry is an important role in the CH_3_SH removal of porous carbon materials, and the Boehm titration method is widely used to evaluated the surface chemistry of activated carbon. Therefore, the chemical analysis of all the porous carbon spheres was completed by means of the Boehm titration, and the corresponding surface pH values and amounts of acidic and basic groups related to the surface of the obtained porous carbon spheres are summarized in Table 2. As can be seen from the Table 2, the number of acidic groups sharply increases for ACSO, and shows the highest value of 1.72 mmol g^−1^ among of all samples. On the contrary, the amount of basic groups nearly decreases to zero (0.02 mmol g^−1^) after thermal air oxidation compared to the virgin carbon spheres ACS. Our previous study has proposed that the reaction between thermal air and the surface of activated carbon spheres was created in the process of air oxidation under 400 °C by means of the surface and pore channels related to the diffusion and etching action, finally resulting in the increase of oxygen containing groups, especially the surface oxygen groups of carbonyl group with strong acidity.^25^ As a result, the larger the number of acidic groups the smaller the pH of the carbon spheres surface. However, when the carbon spheres were subjected to the ammonia heat treatment, no matter direct treatment or preoxidation-assistant modification made a significant contribution to the amount of basic groups for ACSN and ACSON, which can be attributed to the incorporation of basic nitrogen-containing groups by means of decomposition of oxygen containing surface groups and the reaction between the generated free radicals and the surface oxides or active sites.^37,43^ As expected, the pH values and number of basic groups considerably increase compared with ACS and ACSO, and such basic nitrogen-containing on the surface of porous carbon spheres could accounted for the dissociation of CH_3_SH or oxidation of CH_3_S^−^ ions to disulfides. Moreover, these basic groups may take advantage of weak acid–base interactions to attract CH_3_SH. In addition, the amount of acidic groups is almost equivalent to the number of basic groups for CASC with the pH of 7.09.

**Table 2:** Surface pH values, Boehm titration results, amount of preadsorbed water and CH_3_SH breakthrough capacities of all samples

Sample	pH	pHE	Acidic groups (mmol g^−1^)	Basic groups (mmol g^−1^)	Total groups (mmol g^−1^)	Amount of water (mg g^−1^)	CH_3_SH capacity (mg g^−1^)
ACS	7.21	7.03	0.48	0.56	1.04	165.2	181.1
ACS-A	7.21	7.06	—	—	—	0	53.8
ACS-N	7.21	7.08	—	—	—	0	51.2
ACSO	3.03	2.88	3.03	1.72	0.02	76.4	87.7
ACSN	9.13	5.91	9.13	0.46	0.97	169.7	534.3
ACSON	8.62	5.16	8.62	0.42	0.76	172.3	622.8
ACSON-A	8.31	6.57	—	—	—	0	313.9
ACSON-N	8.31	7.24	—	—	—	0	203.6
CACS	7.09	6.63	0.44	0.48	0.92	103.8	132.2

### 3.6 Elemental and XPS analysis

In order to investigate the element contents and the species of the surface functional groups, the prepared carbon spheres were subjected to the Elemental and XPS analysis. As can be seen in Table 3, the carbon and oxygen content show 84.35 wt% and 15.14 wt% for virgin carbon spheres ACS, respectively. However, the carbon content decreases to 74.94 wt% for ACSO, while the oxygen content increases to 23.94 wt% after thermal air oxidation compared to the ACS and CACS. In our study, the resin spheres was firstly activated by steam under 850 °C, and then the obtained carbon spheres were treated with thermal air, in which the diffusion and etching action appeared between the oxygen molecules and the carbon surface or pore channel of activated carbon spheres under 350 °C, including CO_2_ departure, pores expansion or collapse, and the formation of new oxygen containing groups, finally leading to the increase of oxygen containing groups, especially the oxygen content of the oxidized carbon spheres.^25,39^ Furthermore, the virgin and oxidized carbon spheres were separately treated by ammonia thermal treatment, and the reactions between the created free radicals during ammonia decomposition and the surface of carbon spheres considerably introduced nitrogen into the carbon structure. For example, the nitrogen content increased to 5.21 wt% and 7.13 wt% for ACSN and ACSON compared with the ACS (0 wt%), respectively. Moreover, it can be observed that the ACSON shows higher nitrogen content than that of ACSN, which may be ascribe to the method of incorporating nitrogen at high temperatures. The nitrogen content differences between the ACSN and ACSON samples suggested that the oxygen functional groups that existed on the surface of carbon spheres played an important role in the process of ammonia treatment by controlling the amount of nitrogen incorporation to the carbon surface, and preoxidation treatment obviously improved the degree of nitrogen incorporation in the process of ammonia treatment.

Elemental and XPS analysis

**Table:** 

Sample	Elemental analysis (wt%)				XPS analysis (at%)		
	C	H	N	O	C	N	O
ACS	84.35	1.51	0	15.14	83.21	0	16.79
ACSO	74.94	1.12	0	23.94	77.35	0	22.65
ACSN	80.17	1.23	5.21	13.39	78.52	6.14	12.31
ACSON	78.86	1.36	7.13	12.65	80.25	8.02	11.73
CACS	86.38	1.47	0.09	12.06	84.62	0	15.38

**Table:** 

Sample	N content (at%)	N-6	N-5	N-Q	N-X
		398.5 eV	400.2 eV	401.1 eV	403.2 eV
ACSN	6.14	52.16	25.87	16.48	5.49
ACSON	8.02	49.36	26.46	15.73	8.45
CACS	0.09	24.36	49.46	11.37	14.81
ACSON-10	7.87	47.82	28.11	14.05	10.02

In the process of subsequent ammonia thermal treatment, these oxygen containing groups can give rise to thermal decomposition, resulting in the formation of active sites that react with ammonia molecules or free radicals to introduce the nitrogen containing functionalities. For instance, the ammonia molecules react with carboxylic acid sites on the surface of carbon spheres, and ammonium salts are firstly formed. Furthermore, the created ammonium salts transform into amides and/or nitriles groups by dehydration reactions. On the other hand, the reaction of ammonia molecules by the substitution of OH groups can easily create amines on the carbon spheres surface. In addition, the surface oxygen containing groups like ether can be replaced by –NH– in the process of ammonia thermal treatment under high temperature, and imine and finally the pyridine functional groups incorporate on the carbon surface by dehydrogenation reactions. The related reaction equations are as follows.^41,51^

4 R-COO-NH_4_^+^–H_2_O → R-CO-NH_2_–H_2_O → R-C≡N

5 R-OH + NH_3_ → R-NH_2_ + H_2_O

6 R-C=O + NH_3_ → R-C=NH + H_2_O

**Fig. 4:**
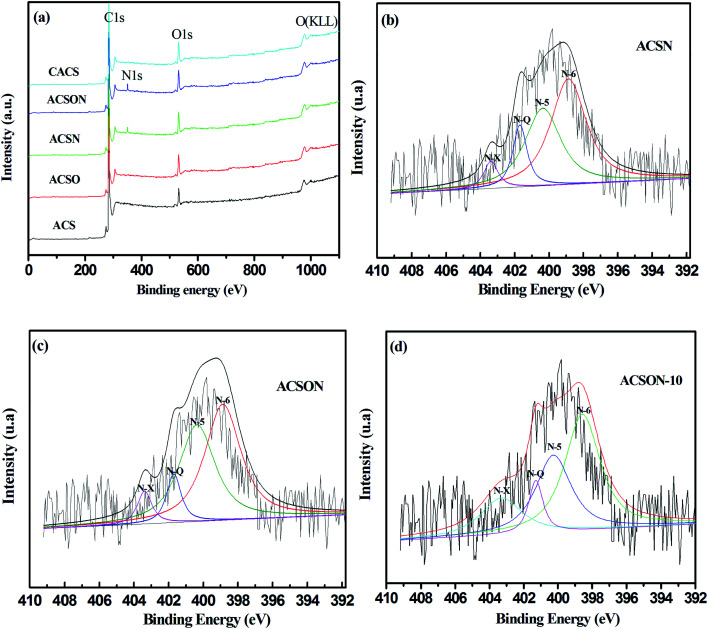
XPS and N 1 s spectra of the activated carbon spheres: (a) survey spectra, (b) ACSN, (c) ACSON and (d) ACSON-10.

In order to investigate the nature of the functional groups created by the above reactions, the elemental speciation and surface binding of the prepared carbon spheres were analyzed by XPS. Fig. 4 shows the XPS spectra of the obtained carbon spheres in this work, it can be seen that the survey spectra of all the adsorbents contain C 1s, O 1s and OKLL spectra (Fig. 4(a)), and it is noticeable that a obvious N 1s peak presents only for ACSN and ACSON, and the envelope N 1s peak of the ACSN and ACSON were consisted of four different bands corresponding to different nitrogen containing functional groups in Fig. 4(b) and (c). For example, the binding energy of 398.7 eV is assigned to pyridinic nitrogen (N-6), the peaks observed at 400.1 eV is attributed to pyrrolic nitrogen (N-5), the binding energy of 401.1 eV is related to quaternary nitrogen (N-Q), and the peaks observed at 403.3 eV is ascribed to pyridine-N-oxide (N-X), respectively.^52,53^ Furthermore, Table 3 summarized the percent contributions of incorporated nitrogen species onto ACSN, CACS and ACSON, and it can be considerably observed that the pyridinic nitrogens are dominant nitrogen species on the surface of ACSN and ACSON. The reason can be attributed to the air oxidation and ammonia treatment, in which thermal air oxidation not only increases the acidic oxygen containing groups, but also gives rise to more active sites on the surface of carbon spheres. Moreover, these created acidic oxygen containing groups and active sites have a profitable contribution to incorporate nitrogen species into the carbon matrix in the type of pyridinic-like nitrogens. In addition, a small amount of pyridine-N-oxide can be created through the transformation of pyridinic nitrogen.^23^ It can be also found that the relative amounts of N-6 and N-Q in the ACSN obtained by direct ammonia treatment show a little higher than those of ACSON prepared by preoxidation-assistant ammonia modification. However, the preoxidation-assistant ammonia modification introduces more nitrogen on the ACSON. As a result, the overall contents of N-6 and N-Q in the ACSON increase when the preoxidation-assistant ammonia modification is adopted for incorporation nitrogen.

### 3.7 CH_3_SH removal performances

**Fig. 5:**
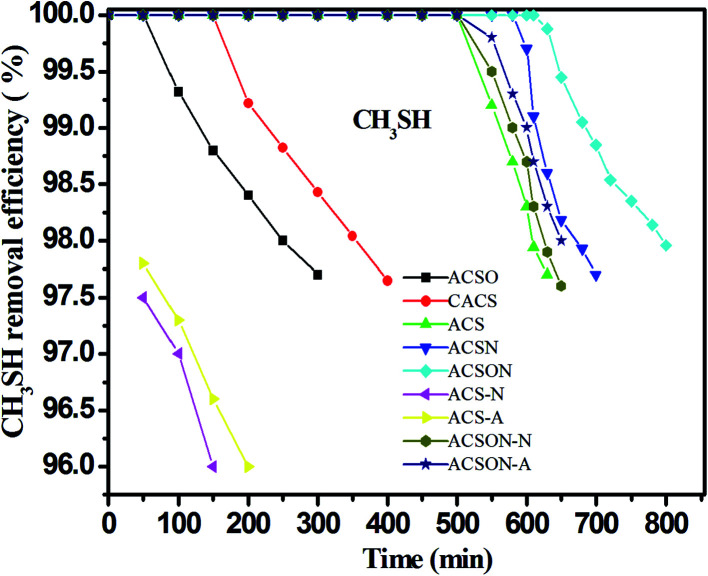
The CH_3_SH removal efficiency of all the samples.

The adsorption/oxidation performances of CH_3_SH over the virgin and modified carbon spheres were evaluated in a fixed bed reactor, and the corresponding breakthrough curves were shown in Fig. 5. It can be observed that the breakthrough time changes accompanied by the nitrogen introduction for all carbon spheres, and the oxidized and commercial carbon spheres show only the breakthrough time of about 200 min, while the preoxidation-assistant ammonia modified carbon spheres present the high breakthrough time of approximately 700 min. The corresponding CH_3_SH breakthrough capacities shown in Table 2 were calculated according to the curves, and it can be seen that the CH_3_SH capacity is 181.1 mg g^−1^ and 132.2 mg g^−1^ for the virgin carbon spheres and commercial carbon spheres, respectively. However, the CH_3_SH breakthrough capacity of ACSO is only 87.7 mg g^−1^, which can be due to the lower surface area and pore volume caused by the air oxidation, especially the negative influence of acidic oxygen species on the surface of ACSO. Furthermore, the higher amounts of nitrogen, the higher CH_3_SH capacities for ammonia modified carbon spheres, and the CH_3_SH capacity is 534.3 mg g^−1^ and 622.8 mg g^−1^ for ACSN and ACSON, respectively. The ACSON shows the highest CH_3_SH capacity with the highest nitrogen content of 7.13 wt%, and the CH_3_SH breakthrough capacity is higher or similar to other reported adsorbents. Therefore, it is critical to introduce the basic nitrogen containing functional groups for enhancement of the CH_3_SH removal.

It has been reported that the two nitrogen containing groups of pyridinic nitrogen and quaternary nitrogen with strong electron transfer ability have a profit for the oxidation of sulfurs species.^21,54^ As can be seen from the XPS analysis, the envelope N 1s peak of the ACSN and ACSON were composed of four different nitrogen containing functional groups, and they presented different relative contents. Thus, the correlation between the CH_3_SH capacities of the ACSN and ACSON and the contents of N-6 and N-Q according to XPS results is also discussed to investigate the effects of the two nitrogen containing species on the CH_3_SH removal as illustrated in the reported literatures. It can be observed that the relative amounts of N-6 and N-Q in the ACSON prepared by preoxidation-assistant ammonia modification show a little smaller than those of ACSN obtained by direct ammonia treatment. However, the preoxidation-assistant ammonia modification introduces more nitrogen on the ACSON with a high nitrogen content of 8.02 at%. As a result, the overall contents of N-6 and N-Q in the ACSON are bigger than those of ASCN, and the CH_3_SH capacity increases with the contents of pyridinic nitrogen and quaternary nitrogen as shown in Fig. 5 and Table 2, which indicates that N-6 and N-Q account for the enhancement of the CH_3_SH oxidation. Therefore, we conclude that the contents of N-6 and N-Q play an important role on the catalytic oxidation of CH_3_SH over carbon spheres.

It has been also essential to study the effect of various gas conditions on the removal of CH_3_SH, thus, the absence of water and oxygen in the mixed gas is implemented for ACS and ACSON, and the results are shown in Fig. 5 and Table 2. It can be seen that the CH_3_SH breakthrough capacity of ACS decreases by around two times under the absence of moisture compared with the presence of moisture. Furthermore, it is noticeable that oxygen has a different effect on the adsorption/oxidation of CH_3_SH for ACS and ACSON. For instance, the CH_3_SH breakthrough capacity is 53.8 mg g^−1^ for ACS-A without moisture, while the ACS-N shows the similary CH_3_SH breakthrough capacity of 51.2 mg g^−1^ in the absence of oxygen. The two conditions indicate that moisture and oxygen have no obvious discrepancy for ACS. On the contrary, the CH_3_SH breakthrough capacity is 313.9 mg g^−1^ for ACSON-A in the presence of dry air, while the ACSON-N has the CH_3_SH breakthrough capacity of 203.6 mg g^−1^ without oxygen. It can be seen that a considerable discrepancy exits in the ACSON. On the one hand, the moisture can create a thin water film on the surface of carbon spheres while the fed gas is in the presence of water, and the adsorbed CH_3_SH can dissolve into the water film, and further dissociate to the thiolate ion that can be easily oxidized to CH_3_SSCH_3_. On the other hand, when the fed gas has no water, the gaseous CH_3_SH is firstly adsorbed onto the surface of carbon spheres, afterwards the adsorbed CH_3_SH is also further oxidized to CH_3_SSCH_3_ in absence of moisture. According to the above results, it can be observed that the CH_3_SH capacity of ACS in dry air and dry nitrogen show little difference, indicating that no oxygen participates in the CH_3_SH removal under air condition, which can be ascribe to the lack catalytic activity sites like nitrogen containing species. However, the CH_3_SH capacity under dry air is bigger than that in dry nitrogen for ACSON, which demonstrates the nitrogen containing functional groups (especially N-6 and N-Q) give rise to the strong catalytic oxidation performance on the surface of ammonia modified carbon spheres. As a result, it can be deduced that water and oxygen have a profit for the CH_3_SH removal over the nitrogen-enriched carbon spheres.

**Fig. 6:**
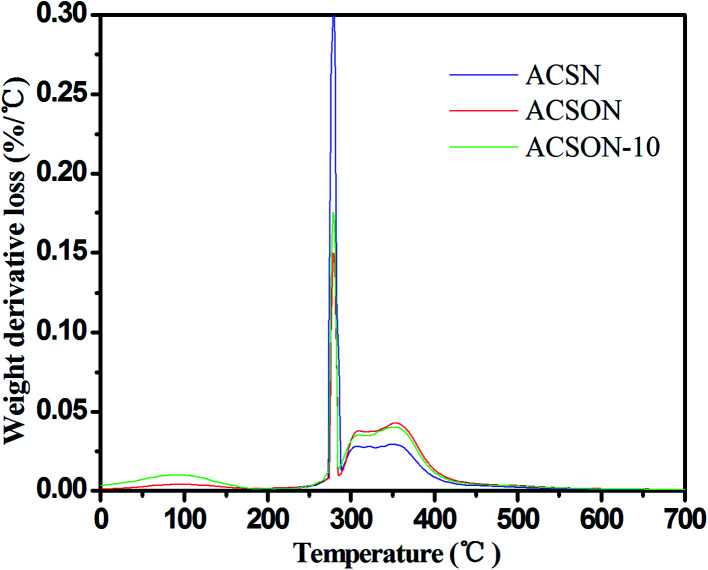
The DTG curves of the exhausted samples.

In order to investigate the created product on the surface of the exhausted carbon spheres, the exhausted samples were subjected to the thermal analysis (TA) experiments, and it can provide the information of related sulfur species through the weight loss in range of certain temperatures.^23,55^ The corresponding DTG curves are shown in Fig. 6 for the exhausted carbon spheres. It can be observed that there are three main peaks for all the samples. For instance, a weak peak at around 90 °C is assigned to the desorption of H_2_O. It has been proposed that the desorption of CH_3_SSCH_3_ created by the oxidation of CH_3_SH is completed in range of 100 °C and 300 °C. Therefore, the sharp peak located at about 280 °C is ascribe to removal of CH_3_SSCH_3_ for the exhausted samples. In addition, another mild peak located between 300 °C and 400 °C is related to the deeper oxidation product, and the peak becomes obvious accompanied by the nitrogen content of the samples.

**Fig. 7:**
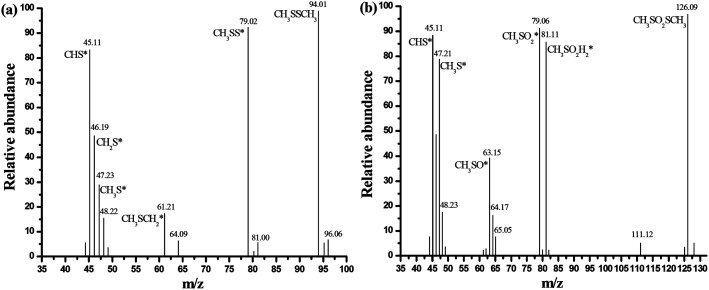
GC/MS spectra for species extracted from ACSON-E: (a) dimethyl disulfide, (b) methyl methanethiosulfonate.

The GC/MS spectra for species extracted from ACSON-E is measured by gas chromatography-mass spectrometry (GC/MS) experiments, and the GC/MS results are shown in Fig. 7. It can be obviously found that the two strong peaks related to the CH_3_SSCH_3_ and methyl methane thiosulfonate appear in the spectra. Combining with the above DTG results, the sharp peak around 280 °C may be also created by the desorption of methyl methane thiosulfonate. It has been observed that further oxidation of sulfur species took place over the ammonia modified carbon spheres under wet air conditions, and the decrease of pH in the exhausted carbon spheres also indicated the formation of acidic oxidation products compared with the fresh samples in Table 2. Therefore, the mild peak located between 300 °C and 400 °C may be caused by the removal of a deeper oxidation product (such as methanesulfonic acid). Furthermore, it is noticeable that the peak area between 250 °C and 300 °C is larger for ACSN than that of ACSON, but the peak area between 300 °C and 400 °C is smaller for ACSN than that of ACSON, which suggest that the more CH_3_SH can be catalytically oxidized by the nitrogen containing active species of ACSON, resulting in the highest CH_3_SH breakthrough capacity. In addition, no peak related to the water is found, indicating that the surface or pore channel of carbon spheres were easily covered by more created CH_3_SSCH_3_ other than water.

**Fig. 8:**
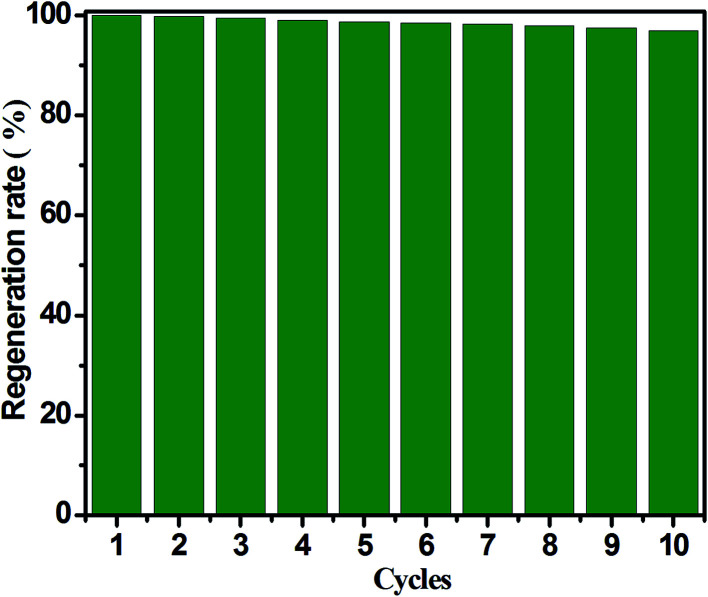
Regeneration cycles of ACSON for CH_3_SH removal.

Another important factor is stable cyclic operation performance relation to the actual application of an adsorbent for CH_3_SH removal, since it can reflects the economy and practicability of porous carbon adsorbents. It is well known that CH_3_SSCH_3_ is the basic oxidation product of CH_3_SH, and it can be easily removed from the surface of carbon adsorbents. Furthermore, the exhausted carbon spheres was regenerated by means of ethanol scrubbing and heat treatment, and Fig. 8 presents the regeneration cycles of ACSON for CH_3_SH removal. It can be observed that no noticeable loss of the CH_3_SH capacity appears for ACSON even after ten cycles, and the CH_3_SH capacity of ACSON reserved as high as 97%. Furthermore, the discrepancies of pore structure and chemical properties were investigated to analysis the cause for fresh ACSON and regenerated ACSON-10. According to the pore structural parameters shown in Table 1, it can be observed that only a slight difference in pore structural parameters is created after ten regeneration cycles. For instance, the BET surface area, total pore volume and microporus volume decreased to 1523 m^2^ g^−1^, 0.69 cm^3^ g^−1^ and 0.56 cm^3^ g^−1^ compared with the fresh ACSON, respectively. And ACSON-10 also keeps a high micropore rate of 81.15%. Moreover, it can be seen that the nitrogen content of ACSON-10 reserves as high as 7.87 at%, and the relative contents of N-6 and N-Q slightly decrease to 47.82% and 14.05%, respectively. Only a slight decrease is shown compared to those of fresh ACSON in Fig. 4 and Table 3. Therefore, it can be inferred that high surface area, large pore volume, narrow pore size distribution with abundant micropores and higher basic surface active sites of porous carbon spheres account for such an excellent CH_3_SH adsorption/oxidation performance.

### 3.8 The CH_3_SH catalytic adsorption/oxidation mechanism analysis

Based on the above results, the possible mechanism is proposed to depict the CH_3_SH adsorption/oxidation over the nitrogen enriched porous carbon spheres. Firstly, a thin water film is formed on the surface of carbon spheres with the introduction of a moist gas, and then CH_3_SH molecules are adsorbed on the surface and dissolve into the water film. Hereafter, they dissociate into protons and thiolate ions. As suggested by Bandosz and Liu, the N-6 (pyridinic nitrogen) and N-Q (quaternary nitrogen) act as the Lewis basic sites and promote the electrons transfer in the adsorption/oxidation of CH_3_SH for the nitrogen doped carbon materials.^21,23^ Afterwards, the dissociation of CH_3_SH to thiolate ions become more easier after the introduction of N-6 with a lone electron pair that play an important role on Lewis basic sites. Later, the extra electrons in the functional groups of N-6 and N-Q, give rise to the transmission between the thiolate ion and the adsorbed oxygen, leading to the formation of thiolate radicals and superoxide ions. Simultaneously, the imported water reacts with the created superoxide ions, and thus hydroxyl radicals are formed in the intermediate process. Next, as the created actives species such as oxygen radicals and hydroxyl radicals also provide benefit conditions for oxidation, the oxidation product of CH_3_SSCH_3_ is further oxidized into CH_3_SO_2_SCH_3_, which is consistent with the results of DTG and GC/MS and other reported literatures,^21,23^ and the oxidation process attains balance states until the oxidation products occupy all the pores with nitrogen active species. Finally, the exhausted carbon spheres are regenerated through ethanol exaction and heat treatment, and the CH_3_SH capacity shows a reserved CH_3_SH capacity of 97% for ten cycles after the slight decrease in BET surface area, total pore volume and nitrogen content of carbon spheres.

## 4. Conclusion

In summary, millimeter resin-based porous carbon spheres with high surface area and microporous structure were firstly prepared from 3-isopropylphenol and formaldehyde *via* suspension polymerization and steam activation, and then the effects of air oxidation and ammonia solution heat treatment on the pore structure and surface chemistry of carbon spheres were investigated for efficient catalytic oxidation of CH_3_SH. The results showed that the obtained porous carbon spheres were mainly microporous, and the carbon spheres showed a high surface area value of 1710 m^2^ g^−1^ and a total pore volume of 0.83 cm^3^ g^−1^ after the direct ammonia treatment. However, the surface area and total pore volume of carbon spheres after air oxidation decreased from 1636 m^2^ g^−1^ to 1397 m^2^ g^−1^ and 0.77 cm^3^ g^−1^ to 0.64 cm^3^ g^−1^ compared with virgin carbon spheres, respectively. Moreover, the preoxidation followed by enriched nitrogen strategy not only increased the surface area and total pore volume of carbon spheres, but also incorporated more active nitrogen species of pyridinic nitrogen and quaternary nitrogen, which demonstrated in this study that the two nitrogen containing functional groups promoted a better catalytic oxidation of CH_3_SH as function of catalysts. Furthermore, the carbon spheres obtained from preoxidation-assisted enriched nitrogen presented the highest CH_3_SH capacity of 622.8 mg g^−1^ with the highest nitrogen content of 7.13 wt%, and the CH_3_SH capacity increased with the contents of pyridinic nitrogen and quaternary nitrogen. In addition, water and oxygen have a profit for the CH_3_SH removal over the nitrogen-enriched carbon spheres. The results of DTG and GC/MS demonstrated that the basic oxidation product is CH_3_SSCH_3_ that can be further oxidized into CH_3_SO_2_SCH_3_. Finally, the exhausted carbon spheres showed a great recycling stability after ten cycles without obvious decrease of the CH_3_SH capacity, which demonstrates that the preoxidation-assisted nitrogen enriched porous carbon spheres are promising for catalytic adsorption/oxidation of CH_3_SH.

## Conflicts of interest

There are no conflicts to declare.

## Supplementary Material

RA-010-D0RA07375J-s001
