# Supplementary material for: Preoxidation-assisted nitrogen enrichment strategy to decorate porous carbon spheres for catalytic adsorption/oxidation of methyl mercaptan
Source: RSC Adv. 2020 Oct 12;10(62):37644–56. doi: 10.1039/d0ra07375j (PMC9057137; doi:10.1039/d0ra07375j)

Electronic Supplementary Material (ESI) for RSC Advances.
This journal is © The Royal Society of Chemistry 2020

Figure captions

Fig.S1. (a) N_2 adsorption-desorption isotherms and (b) DFT pore size distribution .

Fig. S1

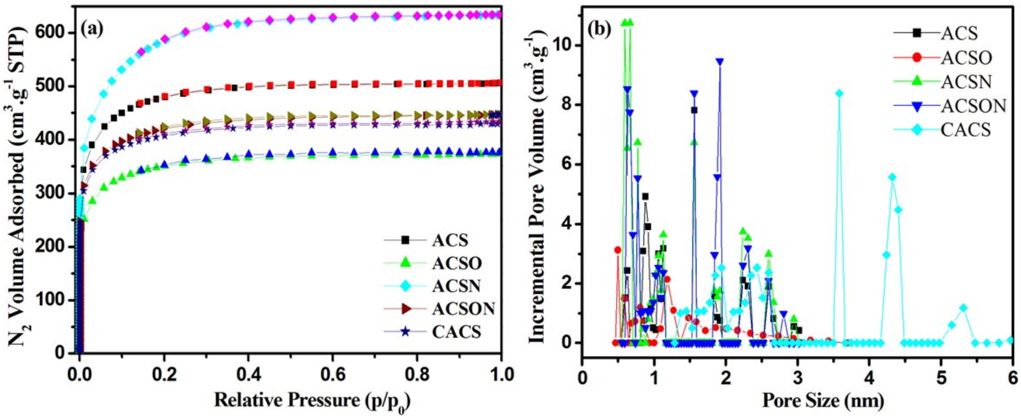

Supplement: RA-010-D0RA07375J-s001 [file RA-010-D0RA07375J-s001.pdf]
